# Optimizing senior’s surgical care - Elder-friendly Approaches to the Surgical Environment (EASE) study: rationale and objectives

**DOI:** 10.1186/s12913-015-1001-2

**Published:** 2015-08-21

**Authors:** Rachel G. Khadaroo, Raj S. Padwal, Adrian S. Wagg, Fiona Clement, Lindsey M. Warkentin, Jayna Holroyd-Leduc

**Affiliations:** 1 Department of Surgery, University of Alberta, Edmonton, AB Canada; 2 Department of Medicine, University of Alberta, Edmonton, AB Canada; 3 Alberta Diabetes Institute, Edmonton, AB Canada; 4 Departments of Medicine and Community Health Sciences, University of Calgary, Calgary, AB Canada; 5 Alberta Seniors Health Strategic Clinical Network, Calgary, AB Canada; 6 2D3.77 Walter C. Mackenzie Health Sciences Centre, 8440-112th Street, Edmonton, T6G 2B7 AB Canada

## Abstract

**Background:**

It is estimated that seniors (≥65 years old) account for >50 % of acute inpatient hospital days and are presenting for surgical evaluation of acute illness in increasing numbers. Unfortunately, conventional acute care models rarely take into account needs of the elderly population. The failure to consider these special needs have resulted in poor outcomes, longer lengths of hospital stay and have likely increased the need for institutional care. Acute Care for the Elderly models on medical wards have demonstrated decreased cost, length of hospital stay, readmissions and improved cognition, function and patient/staff satisfaction. We hypothesize that specific Elder-friendly Approaches to the Surgical Environment (EASE) interventions will similarly improve health outcomes in a cost-effective manner.

**Methods/design:**

Prospective, before-after study with a concurrent control group. Four cohorts of 140 consecutively-screened older patients (≥65 years old) will be enrolled (560 patients in total). The EASE interventions involves co-locating all older surgical patients on a single unit, involving an interdisciplinary care team (including a geriatric specialist) in the development of individual care plans, implementing evidence-informed elder-friendly practices, use of a reconditioning program, and optimizing discharge planning. Subjects will be followed via chart review for their hospital stay, and will then complete in-person or telephone interviews at 6 weeks and 6 months after discharge. Measured outcomes include clinical (postoperative major in-hospital complication or death [primary composite outcome]; death or readmission within 30-days of initial discharge; length of hospital stay), humanistic (quality of life; functional, cognitive, and nutritional status) and economic (health care resource utilization and costs) endpoints. Within-site mean change scores will be computed for the composite primary outcome and the overall covariate-adjusted between-site pre-post difference will be the dependent variable analyzed using generalized linear mixed model procedures including adjustment for clustering.

**Discussion:**

Our findings will generate new knowledge on outcomes from acute surgical care in older patients and validate a novel elder-friendly surgical model including assessment of both clinical and economic benefits. If effective, we expect the EASE initiatives to be generalizable to other surgical centres.

**Trial registration:**

Clinicaltrials.govidentifier:NCT02233153

## Background

### Populations aging and current health care demands

The population of Canada, similar to that of many industrialized nations, is rapidly aging. Seniors (age ≥ 65 years) are anticipated to account for over a quarter of the Canadian population by 2040 [1]. Aging population demographics are expected to cause a marked increase in the prevalence of older patients with complex medical issues, frailty and dependence. Already, it is estimated that 85 % of older adults have at least one chronic health condition [2].

Currently in one province in Canada, seniors account for 63 % of acute inpatient days and 43 % of provincial health expenditure [3, 4]; this experience is common in other jurisdictions. Hospitalized seniors have higher rates of adverse events, surgical complications, and nosocomial infections than younger patients, which contributes to these high costs. They are at higher risk for hospital-acquired delirium, increased length of stay, readmission and loss of capacity to live independently [5–7]. One third of older adults develop new impairments in an activity of daily living during hospitalization and half of these are unable to recover this function [5, 6]. The Canadian health care system needs to adapt health service delivery models in order to meet the demands of our aging population.

### Acute surgery in older adults: challenges specific to this population

It is estimated that more than half of all operations are performed on patients older than 65 years of age. Approximately one third of these patients are discharged to an institutional care facility after major scheduled surgery [8, 9]. Older persons who present to hospital for surgery are often frail and have significant co-existing medical conditions [10], functional and cognitive impairment, limited support mechanisms, and a lower physiological reserve than younger patients. Because older patients are presenting for surgical evaluation of acute illness in increasing numbers, demand for surgical resources is also increasing [10]. Unfortunately, conventional acute care models rarely take into account the specific needs of this population; for example, proactive planning of services such as rehabilitation is seldom done [11]. Acute hospitals continue to be geared to provide care for those with single, acute illnesses rather than those with multiple acute and chronic conditions. Older adults present unique challenges due to comorbidity, cognitive and physical impairment, polypharmacy, and the need for functional rehabilitation and care to support discharge back to the community. Failure to consider these factors can result in poor outcomes, longer lengths of hospital stay and the greater need for institutionalization after surgery. This ultimately results in increased healthcare resource utilization.

Adverse outcomes in this group result from a complex interrelationship between baseline vulnerability and precipitating insults occurring during hospitalization [12]. Currently acute abdominal surgery is accompanied by many such insults that place older persons at particularly high risk for adverse events and functional decline, for example: fasting for gastrointestinal healing, addition of multiple drugs, immobility, nasogastric tubes, and bladder catheterization [12].

Delirium occurs as a common perioperative complication in older patients and is associated with significant adverse outcomes such as increased length of hospital stay, higher postoperative complication rates, falls, mortality and increased discharges to long term care [13, 14]. Delirium is also predictive of poor postoperative functional, cognitive and mobility recovery [15]. Delirium can be prevented using multi-component preventive strategies [14]. Part of this multi-component strategy can include regularly scheduled “comfort” rounds, which address unmet care needs and promote functional independence. Comfort rounds have been shown to improve patient experience in areas of pain management, comfort, and safety, and can reduce falls and pressure ulcers [16–19].

As many as 80 % of older hospitalized patients are either malnourished or at risk of malnourishment, and while in hospital they are at risk for further weight loss, which can lead to increased length of stay, long term care placement and death [20–22]. Adequate perioperative nutrition is of critical importance for patients who are malnourished prior to surgery. Recent studies suggest that earlier postoperative feeding is safe, supports quicker resumption of bowel function and reduces length of stay [23–26]. Functional decline, cognitive impairment and additional factors limiting food intake such as the effects of the underlying illness can further increase the risk for weight loss among older hospitalized patients [27]. Therefore, ensuring adequate access to and intake of food is important and can be achieved through structured processes that involve appropriate assistance with meals and ensuring adequate access to food and fluids [28, 29].

### Traditional surgical care vs. Elder focused surgical care

Standard hospital care can be risky for older people [12, 30–33] and is designed to deal with acute illness with expediency. Acute care surgery (ACS) is a surgical specialty that encompasses trauma and acute surgical disease (e.g. appendicitis, gallbladder inflammation, gastrointestinal obstruction, perforation and emergency cancer surgery) with the goal of providing optimal surgical care within the first 24 h of hospitalization. The ACS model has been embraced by many acute care sites as a means to decrease waiting times in the emergency department, as well as to improve the timeliness of surgery and operational efficiencies [34–36]. However, current ACS service delivery models do not take into account the unique characteristics and needs of the older population.

While specialized care for acutely ill older persons is not new, there is little evidence supporting models of geriatric care until the Acute Care for the Elderly model [37–39]. Originally developed to curb preventable functional decline experienced by older patients admitted to acute medical hospital wards, the Acute Care for the Elderly model emphasizes: (1) a specialized environment, (2) patient-centred care, (3) medical review, and (4) interdisciplinary team plans of care. The objective of elder-friendly hospital care was founded on gerontological principles such as screening for early identification of geriatric syndromes, family and caregiver involvement at all stages of care, interdisciplinary assessments, a holistic focus, respect for choices, and an environment supportive of discharge planning and community services [31]. Compared to traditional care, Acute Care for the Elderly models on medical wards have demonstrated decreased cost, length of hospital stay, readmissions, and improved cognition, function and patient/staff satisfaction [38–40]. To our knowledge, no prior study has developed an Acute Care for the Elderly model for elderly acute surgical patients.

For the reasons outlined above, studies assessing innovative ways to optimize care delivery in elders undergoing acute care surgery are urgently needed [41]. This is the first study to examine the impact of a novel care delivery redesign – Elder-Friendly Approaches to the Surgical Environment (EASE) - in elders receiving acute care surgery.

### Aim & objectives

The aim of the EASE study is to assess the impact of an elder-friendly surgical unit, with the goal of delivering evidence-informed care to improve the quality of care to older acute surgical patients, in order to reduce unnecessary health care resource utilization. The EASE study will realign current resources, implement evidence-informed practices, and improve health outcomes in a cost-effective manner.

The specific objectives of the EASE Study are to:

1. Assess the clinical effectiveness of the EASE initiatives in reducing the rate of post-surgical complications, in-hospital mortality and length of stay, compared to controls.
2. Determine if the EASE initiatives improves humanistic outcomes including health-related quality of life (HRQL), functional capacity and patient satisfaction, compared to controls.
3. Determine the incremental cost and cost-effectiveness of the EASE initiatives.

In aggregate, these objectives will assess the program’s impact on a comprehensive range of outcomes important to patients, providers, and policy-makers.

## Methods

### Study design

This is a prospective, before-after study with a concurrent control group. Four cohorts of consecutively-screened older patients (≥65 years old) will be included:

Intervention Site (University of Alberta Hospital, Edmonton, Alberta Canada)

(i) Hospitalized on the ACS service “before” or pre-EASE initiatives (140 participants)
(ii) Hospitalized on the ACS service “after” or post-EASE initiatives (140 participants)

Control Site (Foothills Medical Centre, Calgary, Alberta, Canada)

(iii) Hospitalized on the ACS service pre- EASE initiatives (140 participants, same enrollment period as (i) above)
(iv) Hospitalized on the ACS service post- EASE initiatives (140 participants, same enrollment period as (ii) above)

Cohorts iii, and iv will serve as concurrent controls since the EASE initiatives will not have been implemented in that hospital. Of note, the ACS services at the intervention and control sites are structured similarly and admit similar patients.

### Subjects

Inclusion criteria (all criteria must be met):

1. All patients ≥ 65 years old
2. Index admission to ACS Service
3. Postoperative acute abdominal surgery

Exclusion criteria (any one sufficient to exclude):

1. Elective general surgery cases
2. Palliative surgery cases (surgery with the primary intention of improving quality of life or relieving symptoms caused by advanced non-curable disease)
3. Trauma surgery cases
4. Non-abdominal emergency surgery cases
5. Nursing home resident requiring full nursing care on admission (dependency in 3 or more activities of daily living)
6. Patients from out of province, or transferred from another in-hospital inpatient service

### Interventions

The EASE initiative will involve:

- **Capacity re-alignment:** elderly surgical patients are currently scattered across different nursing units. Patients will be co-located on 1 unit to allow better implementation of the initiatives and coordination of care.
- **Interdisciplinary care delivery:** will include daily ward rounds by geriatric specialists, in addition to consultation with rehabilitation providers, pharmacists, dieticians and social workers within 24 h of admission to help in the development of individual care plans.
- **Implementation of evidence-informed practices:** optimizing evidence-based guideline adherence, such as:
- Medication review and reconciliation
- Early mobilization on the surgical unit
- Prevention of postoperative complications (e.g. venous thromboembolism prophylaxis)
- Incorporation of Enhanced Recovery After Surgery program principles such as early feeding following surgery, reduced use of total parenteral nutrition, and avoidance or early removal of tubes [nasogastric, urinary catheters], drains, and lines [intravenous, central lines] [42].
- Elder-friendly practices such as comfort rounds, performed every 2 h by the nursing staff to address unmet care needs, mobility, nutrition, pain management, and prevention of falls and delirium.
- Health care provider/patient/family education
- **Reconditioning program:** a targeted, functional program that patients can perform independently at their bedside, beginning immediately post-operatively.
- **Transition optimization:** the interdisciplinary teams (from 2 above) will define the optimal blend of patient-specific services required to support safe, quality transitions to discharge with a goal to maximize functional recovery and return to baseline health status. This coordinated plan will be established at admission and will include appropriate discussions with the patients and caregivers.

### Data collection

Study personnel will collect data using standardized case report forms recorded on an electronic database using a secure, internet-based portal.

1. **Socio-demographic information (baseline)**: including age, sex, ethnicity, body mass index, medical comorbidities, residence prior to admission, blood pressure, heart rate, respiratory rate, haemoglobin, creatinine and medication history. Data obtained from chart review will be used to create the Charlson comorbidity index [43] and Rockwood’s frailty index [44].
2. **Hospitalization details (baseline):** including admitting diagnosis, operative procedure, operating surgeon, American Society of Anaesthesiologists Class, requirements for alternative service consults, total parenteral nutrition and urinary catheter utilization, discharge location, and any new requirement for alternative level of care upon discharge.
3. **Major in-hospital complications (baseline):** all complications are defined using the Centers for Disease Control definitions [45], including:
  - Intensive care unit admission (includes respiratory failure, cardiac arrest or septic shock),
  - Vascular complications (myocardial infarction, stroke, deep venous thrombosis, pulmonary embolism)
  - Serious infections (pneumonia, intra-abdominal abscess, urinary tract infection, deep wound infection or infected decubitus ulcer), and
  - Protracted delirium (≥48 h). A validated chart-based method for identification of delirium will be utilized [46]. This chart-based instrument has a sensitivity of 74 % and a specificity of 83 %, using the Confusion Assessment Method [47] as the gold standard.
4. **Mortality (baseline, 6 weeks, 6 months):** divided into in-hospital mortality, 30-day mortality and 6 month mortality.
5. **Length of hospital stay (baseline):** divided into:
  - acute length of stay defined as time until the acute medical treatment for that patient is complete;
  - total length of stay which includes both medical treatment time and time awaiting transfer to another hospital (e.g., rural hospital) or alternative level of care (assisted living, long-term care placement or rehabilitation).
6. **Readmission (6 weeks, 6 months):** divided into 30-day readmission and 6 month readmission.
7. **Functional status (6 weeks):** including:
  - Edmonton Frail Scale [48]: a valid and reliable tool to assess the frailty of the older patients. The scale has 11 items assessing cognition, general health status, functional independence, social support, medication use, nutrition, mood, continence and functional performance. Higher scores indicate greater frailty.
  - Timed Up and Go [49]: a commonly used screening tool for falls risk. The patient is timed while they rise from an arm chair, walk three metres at a comfortable and safe pace, turn and walk back to the chair and sit down again. Faster time indicates a better functional performance.
8. **Cognitive status (6 weeks):** using the Abbreviated Mental Test Score [50]. This 10 item test screens older patients for possible cognitive impairment, with lower scores indicating greater impairment.
9. **Quality of life and health status (6 weeks and 6 months):**
  - EQ-5D [51]: A widely used, validated, preference based generic HRQL instrument. It encompasses five domains of HRQL (mobility, self-care, usual activities, pain/discomfort, and anxiety/depression) and a visual analogue scale. The five health domains can be scored and totalled to yield a single Index (EQ-Index) score anchored between 0 (death) and 1 (full health). The visual analogue scale ranges from 0 to 100 with higher scores indicating better HRQL. The EQ-5D is also a measure of utility; a requirement for cost-effectiveness analyses.
  - SF-12 [52]: A widely used, validated, generic HRQL instrument. It yields a physical and a mental health component summary score. Higher scores indicate better health status.
10. **Nutritional status (6 weeks and 6 months):** using the Malnutrition Screening Tool [53]. This is a brief screening tool to assess nutritional status, with smaller scores indicating better nutritional status.
11. **Health care resource utilization and costs (baseline and 6 months):** Health care resource utilization will be assessed through financial micro-costing databases. A modified health resource utilization questionnaire [54] will be administered at the final follow up visit to identify patient out-of-pocket expenses and indirect carer costs.

### Outcomes

The primary outcome from this study is the proportion of patients who experience a postoperative major in-hospital complication or death (composite outcome). Secondary outcomes include: the proportion of patients who experience each component of the primary composite outcome (in-hospital mortality, intensive care unit admission, vascular complications, serious infections, protracted delirium); the proportion of patients who die or are readmitted within 30-days of initial discharge; length of hospital stay; discharge location; requirement for an alternative level of care upon discharge; quality of life; functional, cognitive and nutritional status; and health care resource utilization and costs.

### Data analysis

We will evaluate the effectiveness of the EASE initiative implementation by comparing between-site mean changes in each outcome in the cohort of patients hospitalized from the baseline cohort (defined as “pre-EASE”) to those hospitalized after implementation (defined as “post-EASE”).

Initially, exploratory data analysis will be performed on each major variable, including calculation of means, medians, proportions, and measures of central tendency. Baseline variables pre-EASE and post-EASE will be compared between sites using *X*^2^ tests for categorical variables, Student T-tests for normally distributed continuous variables, and the Wilcoxon rank sum test for non-normally distributed variables.

Secondly, within-site mean change scores will be computed for the composite primary outcome (difference of proportions) and the overall covariate-adjusted between-site pre-post difference (‘difference of differences’) will be the dependent variable analyzed using generalized linear mixed model procedures and will include adjustment for clustering by study site and operating surgeon. The regression model will be constructed using recommended techniques and assessment of model assumptions (including tests of normality, tests of colinearity and log-transformation of non-normally distributed continuous variables) will be performed as necessary. Additional adjustment for baseline age, sex, BMI, ethnicity, blood pressure, heart rate, respiratory rate, haemoglobin, creatinine, admitting diagnosis, medical comorbidities, operative procedure, Rockwood clinical frailty score, Charlson comorbidity index, American Society of Anaesthesiologists class, baseline month of enrolment, and/or baseline place of residence will be performed. Secondary endpoints will be analyzed as ‘difference of differences’ in a manner similar to that outlined for the primary outcome.

For cost analysis, exploratory baseline analyses will be completed including calculation of mean index hospitalization cost, median index hospitalization cost, mean readmission cost, median readmission cost and measures of central tendency for both variables. The cost of the intervention will also be calculated and included in the total cost post-EASE. All costs pre-EASE and post-EASE will be compared between sites. The difference in costs between sites will be calculated and compared. If required, a risk-adjustment model to predict cost differences between sites will be developed. A cost per quality adjusted life year (QALY) gained will also be calculated. The mean cost difference in each site will be divided by the QALYs achieved in each site. QALYs will be calculated using the utility values measured by the EQ-5D and the length of life of each patient.

### Sample size calculation

Based on our pilot data, we anticipate the incidence of the primary composite outcome to be 35 % pre-EASE (cohorts (i) and (iii)) [55–57]. A 10 % absolute difference in the primary endpoint is considered to be the minimal clinically important difference to detect (a commonly used threshold for many interventions [58]). Accordingly, assuming an alpha = 0.05, beta = 0.2 (80 % power), a 15 % decline in the primary outcome post-EASE, a 5 % decline in the primary outcome in the control group (10 % absolute overall ‘difference of differences', 1:1 recruitment of cases to controls), we will require 140 patients per centre (UAH, Edmonton and FMC, Calgary) or 280 patients total. Since all enrolled patients will be followed by chart review to ascertain the primary outcome, there will be no drop-outs and no sample size adjustment will be required for attrition. However, a priori, we have decided to double the required sample size (280 patients per site or 560 in total) to account for the likelihood of high attrition at follow up. Given the absence of previous studies, after collecting data on the first 140 patients (total of both sites), we will calculate an updated sample size calculation.

### Ethical and other considerations

The EASE study protocol has received approval from the University of Alberta Research Ethics Board (Pro00047180) and the University of Calgary Conjoint Research Ethics Board (REB140729). The trial has received peer reviewed funding from Alberta Innovates Health Solutions (grant # 201300465) and has been formally registered at clinicaltrials.gov (NCT02233153).

An exemption from patient consent at baseline (in-hospital stay via chart review) has been granted, as patients will be acutely ill and will require sedatives and pain killers making obtaining informed consent at that stage impossible. Signed informed consent will be obtained prior to hospital discharge so that in-person and telephone follow-up interviews can be performed, 6 weeks and 6 months post discharge.

## Discussion

In summary, EASE is a prospective, before-after study with a concurrent control group. EASE aims to assess the impact of an elder-friendly surgical unit with the goal of delivering evidence-informed care to improve the quality of care to older acute surgical patients and result in optimal use of health care resources.

Given the current aging population and the rising burden of chronic disease and frailty, new patient-centred, comprehensive and interdisciplinary models of Acute Care Surgery are needed to optimize care in this vulnerable patient population. This proposed research will generate new knowledge on acute surgical outcomes in older patients and validate the novel model of care, including assessment of the associated economic benefits. If effective, we expect the EASE initiatives to be generalizable to other surgical centers.

EASE commenced enrolment in April 2014. Recruitment of all pre-EASE subjects is expected by spring 2015, with implementation of the EASE initiatives in summer 2015. Completion of all post-EASE subject recruitment is expected to take until mid-2016, with final study results anticipated in late 2016.

## Abbreviations

ACS: Acute care surgery
EASE: Elder-friendly approaches to the surgical environment
HRQL: Health-related quality of life
QALY: Quality adjusted life year
